# Urinary Neutrophil Gelatinase-Associated Lipocalin Is Complementary to Albuminuria in Diagnosis of Early-Stage Diabetic Kidney Disease in Type 2 Diabetes

**DOI:** 10.1155/2017/4691389

**Published:** 2017-08-06

**Authors:** Agnieszka Gala-Błądzińska, Paulina Dumnicka, Beata Kuśnierz-Cabala, Katarzyna Rybak, Ryszard Drożdż, Agnieszka Żyłka, Marek Kuźniewski

**Affiliations:** ^1^Department of Dialysis and Nephrology, St. Queen Jadwiga Clinical District Hospital No. 2 in Rzeszów, Lwowska 60 Street, 35-301 Rzeszów, Poland; ^2^Department of Medical Diagnostics, Jagiellonian University Medical College, Medyczna 9, 30-688 Kraków, Poland; ^3^Department of Diagnostics, Chair of Clinical Biochemistry, Jagiellonian University Medical College, 15A Kopernika Street, 31-501 Krakow, Poland; ^4^Department of Internal Medicine, Nephrology & Endocrinology, St. Queen Jadwiga Clinical District Hospital No. 2 in Rzeszów, Lwowska 60 Street, 35-301 Rzeszów, Poland; ^5^St. Queen Jadwiga Clinical District Hospital No. 2 in Rzeszów, Lwowska 60 Street, 35-301 Rzeszów, Poland; ^6^Department of Nephrology, Jagiellonian University Medical College, 15 Kopernika Street, 31-501 Krakow, Poland

## Abstract

**Background:**

Two clinical phenotypes of diabetic kidney disease (DKD) have been reported, that is, with or without increased albuminuria. The aim of study was to assess the usefulness of urinary neutrophil gelatinase-associated lipocalin (uNGAL) for the early diagnosis of DKD in the type 2 diabetes mellitus (T2DM).

**Methods:**

The study group consisted of 123 patients with T2DM (mean age 62 ± 14 years), with urine albumin/creatinine ratio (uACR) < 300 mg/g and eGFR ≥ 60 ml/min/1.73 m^2^. The control group included 22 nondiabetic patients with comparable age, sex, and comorbidities. uNGAL, albumin, and creatinine were measured in the first morning urine samples. uACR and uNGAL/creatinine ratios (uNCR) were calculated.

**Results:**

In the control group, maximum uNCR was 39.64 *µ*g/g. In T2DM group, 24 patients (20%) had higher results, with the maximum value of 378.6 *µ*g/g. Among patients with uNCR > 39.64 *µ*g/g, 13 (54%) did not have markedly increased albuminuria. Women with T2DM had higher uNCR than men (*p* < 0.001), without difference in uACR (*p* = 0.09). uNCR in T2DM patients correlated significantly with HbA1c. Sex, total cholesterol, and uACR were independent predictors of uNCR above 39.64 *µ*g/g.

**Conclusions:**

Increased uNGAL and uNCR may indicate early tubular damage, associated with dyslipidemia and worse diabetes control, especially in females with T2DM.

## 1. Introduction

Diabetes is a group of metabolic diseases characterized by heterogenic pathophysiology and clinical manifestations. Type 2 diabetes (T2DM) is the most frequent type of carbohydrate metabolic disorders; it is estimated that T2DM comprises 90–95% of all cases. In most countries, T2DM prevalence is constantly increasing, and the increase is faster than the population growth [1]. In addition, a major medical problem is the increasing morbidity and mortality from complications of diabetes, affecting eyes, kidneys, heart, cardiovascular, and nervous system [2]. In most countries, the diabetic kidney disease (DKD) is the most common cause of nephropathies requiring renal replacement therapy [3]. Morphological changes in kidneys in diabetes are induced by disorders of kidney metabolism caused by increased glycemia, as well as by changes in renal hemodynamics, or activation of the renin-angiotensin-aldosterone system (RAA). In a substantial proportion of T2DM patients, structural changes in kidneys as well as the structural-functional relationships differ from the classical Kimmelstiel-Wilson nodular sclerosis observed in type 1 diabetes (T1DM) [4]. In particular, histopathological studies suggest tubular involvement in about 40% of patients with DKD associated with T2DM, and tubular changes in these patients are unproportionate comparing with glomerular pathology [4–6]. Literature provides convincing evidence that changes in kidneys of T2DM patients are more heterogenic than in T1DM [4–7].

Neutrophil gelatinase-associated lipocalin (NGAL) was first identified in activated neutrophils. It belongs to the lipocalin protein family, and it is able to bind and transport small ligands [8]. Under physiological conditions NGAL is expressed at very low levels in kidneys, trachea, lungs, stomach, and colon. It is present in blood in low concentrations and it undergoes free glomerular filtration followed by nearly complete resorption in the mechanism of megalin-mediated endocytosis in the renal proximal tubule. Increased NGAL synthesis in response to a damaging factor in the distal convoluted tubule and urinary secretion of NGAL constitute the major fraction of urinary NGAL [9, 10]. NGAL as an early biomarker of kidney damage was identified in 2003 during studies searching for novel markers of ischemic and toxic kidney injury in patients undergoing cardiac surgery [9]. The urinary concentrations of NGAL (uNGAL) increase in a consequence of tubular dysfunction associated with acute kidney injury caused by ischemia and secondary tubular damage [9]. Studies suggest that uNGAL may be an appropriate biomarker of tubular changes in chronic kidney disease including DKD, both in T2DM and in type T1DM [11–13]. The studies of Fu et al. [14] and Kim et al. [15] suggested important role of uNGAL measurements in early diagnosis of DKD. Among patients with T2DM, an increase in uNGAL significantly correlated with a decrease in GFR [11, 16].

The aim of the study was to assess the function of renal tubules in patients with early-stage T2DM as reflected by uNGAL concentrations in a group of T2DM patients at the early stage of DKD, that is, with eGFR ≥ 60 ml/min/1.73 m^2^ and urine albumin/creatinine ratio (uACR) < 300 mg/g.

## 2. Materials and Methods

The study was conducted in accordance with the Declaration of Helsinki and received permission from the Bioethics Committee of the Regional Medical Chamber in Rzeszów, Poland (number 70/2014/B).

The study recruited adult patients with T2DM who were referred to the ambulatory specialist nephrological care by their diabetologist. Between 2014 and 2015, 123 patients were enrolled in the study. Inclusion criteria were eGFR (2009 Chronic Kidney Disease, Epidemiology Collaboration, CKD-EPI equation) >60 ml/min/1.73 m^2^ and no overt proteinuria (uACR < 300 mg/g). Only patients who signed the informed consent were included in the study. Exclusion criteria were treatment with nephrotoxic medications, other kidney diseases, urinary tract infections, systemic infections, cancer, allergy, systemic connective tissue diseases, anemia, pregnancy, and nonstable hypertension (≥130/90 mmHg in self-monitoring). Additionally, a control group included 22 nondiabetic patients with age, sex, and comorbidities similar to T2DM patients; this allowed comparison of laboratory results. The control group included adult patients of the nephrological ambulatory with eGFR CKD-EPI > 60 ml/min/1.73 m^2^ and no overt proteinuria. These were mainly patients with stable arterial hypertension, or benign simple kidney cysts. Two control patients suffered in the past infections of the lower urinary tract; one patient was diagnosed with duplication of renal pelvis. The exclusion criteria were the same as for T2DM patients.

First morning urine samples were taken from patients and controls for the measurements of uNGAL, albumin, and creatinine concentrations, as well as for the general urine examination. The concentrations of uNGAL were measured with the automated chemiluminescent microparticle immunoassay on the ARCHITECT analyzer (Abbott Diagnostics, Abbott Park USA). Urine albumin was measured with immunoturbidimetry and urine creatinine with enzymatic method using Olympus AU680 biochemistry analyzer (Olympus, Center Valley, PA, USA). The results of the measurements were used to calculate uACR and uNGAL/creatinine ratio (uNCR). Other laboratory results were obtained as a part of routine patients' assessment performed in nephrology ambulatory.

### 2.1. Statistical Analysis

A number of patients (percentage of the group) are reported for qualitative variables. Mean ± standard deviation or median (lower-upper quartile) are shown for normally or nonnormally distributed quantitative variables, respectively. The distributions of the variables were assessed with Shapiro-Wilk's test. The *t*-test or Mann-Whitney's* U* test was used to study differences between the groups. The variables that differed significantly between patients with high and low uNCR were used as predictor variables in multiple regression analysis. Odds ratios with 95% confidence intervals were reported for multiple regression analysis. Spearman's correlation coefficients are reported for correlations. All the tests were two-tailed and the results at *p* < 0.05 were considered statistically significant.

## 3. Results

The group of T2DM patients did not differ from the control group participants in terms of age, sex, eGFR, or cardiovascular comorbidities, but they had higher BMI (Table 1). Also, the average concentrations of urine albumin and NGAL, as well as the values of uACR and uNCR, did not differ between diabetic patients and control subjects (Table 1). However, the maximum uNCR in the control group was 39.64 *µ*g/g and, among studied T2DM patients, 24 (20%) had higher values, with the maximum of 378.6 *µ*g/g.

**Figure 1:**
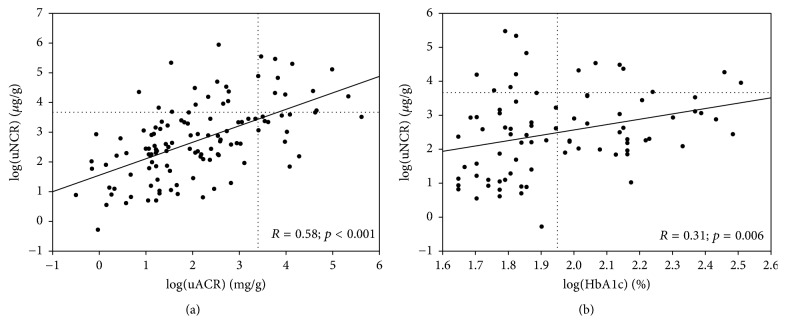
The associations between uNCR and uACR (a) and HbA1c (b) among T2DM patients. The reference lines denote uNCR = 39.64 *µ*g/g (i.e., maximum in the control group), uACR = 30 mg/g, and HbA1c = 7%.

T2DM patients with uNCR above the maximum control value were characterized by higher triglycerides, total cholesterol, and LDL-cholesterol, as well as higher urine albumin and uACR as compared to patients with lower uNCR (Table 2). The correlation between uNCR and uACR was highly significant (Figure 1(a)). Still, in 13 (54%) of the 24 patients with high uNCR values, uACR was below 30 mg/g and ranged from 2.35 to 16.10 mg/g. Interestingly, the patients with high uNCR were mainly women (*N* = 21, i.e., 88%). Women with T2DM had significantly higher uNCR than men [24.23 (8.89–56.80) versus 11.40 (3.36–18.02) *µ*g/g; *p* < 0.001], without significant difference in uACR [8.87 (3.41–33.45) versus 5.33 (3.15–13.28) mg/g; *p* = 0.09]. The average concentrations of uNGAL were also higher in DMT2 women than men, although the difference was not statistically significant [17.15 (7.60–43.90) versus 13.70 (6.10–23.80) *µ*g/l; *p* = 0.1].

Among 123 T2DM patients, 94 (76%) underwent the ophthalmologic examination, including 83 with low uNCR and 11 with high uNCR. We did not observe significant associations between uNCR and the presence of diabetic retinopathy (Table 2). Age, eGFR, and BMI values, as well as known diabetes duration, did not differ between the groups with uNCR above and below 39.64 *µ*g/g (Table 2). Although HbA1c was significantly correlated with uNCR (Figure 1(b)), it did not differ significantly between the patients with uNCR above and below the maximum control value (Table 2). uNCR did not correlate with eGFR (*R* = −0.14; *p* = 0.1), age (*R* = 0.14; *p* = 0.1), or time from T2DM diagnosis (*R* = 0.13; *p* = 0.1). uNCR was nonsignificantly higher among patients treated with angiotensin-converting enzyme inhibitors (ACEI) or angiotensin receptor blockers (ARB) comparing with those not consuming the medications [15.87 (7.90–36.02) versus 9.72 (3.82–23.70) *µ*g/g; *p* = 0.053], while uACR did not differ between the groups [7.89 (3.37–18.40) versus 4.58 (3.05–16.90); *p* = 0.4]. In multiple logistic regression (Table 3), sex, total cholesterol, and uACR were identified as the independent predictors of high uNCR (i.e., above the maximum control value of 39.64 *µ*g/g).

In control group, no significant correlation was observed between uNCR and uACR. Also, there were no significant differences between control men and women in uNGAL and uNCR values. In contrast to DMT2 patients, uNGAL concentrations were nonsignificantly higher in control men [20.10 (7.40–48.40) versus 10.50 (6.00–20.30) *µ*g/l; *p* = 0.1]; however, uNCR was nonsignificantly higher in control women [12.53 (6.42–28.81) versus 7.73 (5.92–13.69) *µ*g/g; *p* = 0.3].

## 4. Discussion

In most cases, recognition of the diabetic kidney disease (DKD) is based on results of tests such as albuminuria, creatininemia with the estimation of eGFR, or renal imaging. Clinical symptoms of DKD appear late and are not characteristic. In T1DM, DKD coexists with diabetic retinopathy that can be detected in ophthalmologic examination. However, in T2DM, DKD may be present in patients without retinopathy [17, 18]. Diabetic retinopathy is less frequent in T2DM and is a poor predictor of type of nephropathy [18]. Our results are consistent with these observations, as we did not observe significant associations between uNCR and the presence of retinopathy. However, our observations regarding retinopathy must be treated with caution, as only a part of patients underwent ophthalmologic examination.

Renal biopsy is performed only in a relatively small number of T2DM patients. This usually happens during advanced stages of the disease when serum creatinine is elevated and overt proteinuria occurs. In T2DM patients, the morphological abnormalities in kidneys and the clinical course of DKD are varied. In this group of patients, not only classical glomerular changes but also changes in renal tubules and in the renal interstitium play an important role in kidney failure [5, 6, 19]. In nearly 40% of T2DM patients, renal biopsy does not reveal typical glomerular pattern as observed in T1DM. In a substantial proportion of such patients, biopsy results show disproportionately severe damage to the tubulointerstitial tissue as well as hyaline changes in small renal arteries [5, 6]. For these reasons, eGFR and albuminuria (proposed by Kidney Diseases Improving Global Outcomes initiative [20] for the clinical assessment and prediction of CKD progression) may be insufficient in the early assessment of kidney function among T2DM patients. If albuminuria and uACR, together with eGFR, are considered markers of glomerular damage, then in the group of T2DM in our study the occurrence of CKD stages G1, G2, and A1 can only be suspected; we can, however, recognize stages G1, G2, and A2. In turn, when the uNCR above 39.64 *µ*g/g (i.e., the maximum uNCR in the control group) is considered a marker of tubular and interstitial damage, a subgroup of 20% of patients with tubular damage can be distinguished. This tubular damage cannot be discovered during routine nephrological diagnostic tests. More than half of the patients in our study had normal uACR values according to the current diagnostic criteria [20], that is, uACR lower than 30 mg/g. In such patients, a clinicist may not become alert enough to be able to recognize early nephropathy connected with T2DM. Our study shows that especially in women with T2DM with abnormal lipid profile and inadequate diabetes control the diabetic kidney disease may be underdiagnosed. The United Kingdom prospective diabetes study (UKPDS) [21] indicates that in women with T2DM the decrease in glomerular filtration is frequently not accompanied by albuminuria. Similarly, in a study of Parving et al. [22], more than 50% of patients had no albuminuria. The study analyzed data from more than 24000 T2DM patients, nearly 80% of whom had glomerular filtration above 60 ml/min/1.73 m^2^ [22].

Higher uNCR values in women with T2DM are partly due to lower urine creatinine excretion in women than in men. We have observed this both in T2DM patients and in controls. However, control women had lower uNGAL concentrations than control men while, among T2DM patients, uNGAL was higher in women than in men. Thrailkill et al. [23] observed higher uNGAL concentrations in females compared to males in subjects with T1DM [23]. Higher uNCR values in women with T2DM suggest that the early stages of DKD may be similarly common in both sexes or even more common in women, although end-stage renal disease is in fact more common in diabetic men [24–26]. Female gender is protective against the development of end-stage renal disease in nondiabetic renal disease [24, 27] but this gender-protective effect is probably diminished in diabetes mellitus [28, 29].

In our study, uNCR correlated positively with uACR in the T2DM patients. This is consistent with the results of Nielsen et al. [30], who observed correlation between uNGAL and albuminuria among 177 patients with T2DM and normal eGFR during 3.5 years of follow-up. Increased uNGAL predicted the increase in urinary albumin excretion ranging from “microalbuminuria” to “macroalbuminuria” and higher concentrations of uNGAL were associated with a more rapid deterioration of renal function [30]. Our study indicates that patients with uNCR above 39.64 *µ*g/g had on average higher albuminuria and uACR. Several pathomechanisms may be listed as underlying this observation. If we assume DKD with primary glomerular involvement, the increased urinary excretion of NGAL may result from disrupted mechanisms of protein transport involving megalin and cubilin, caused by long-term, excessive reabsorption of albumin in tubules [31, 32]. Also, other substances that leak to primary urine through the damaged glomerular barrier may cause tubular cells' damage, hence initiating inflammation and the process of renal interstitial fibrosis. This, in turn, contributes to further kidney damage resulting in albuminuria cooccurring with tubular proteinuria and increased urinary excretion of NGAL [33]. However, as clearly indicated in practice guidelines on DKD [34], nephropathy other than early glomerular damage may be responsible for the increased urinary excretion of NGAL in diabetic patients. Hence, in our study, the increase in uNCR in the group of patients with normal albuminuria may also be linked with primary tubular damage. In diabetic patients, tubular cells are negatively affected by hyperglycemic environment. This leads to the development of inflammation in the tubulointerstitial tissue, increased production of extracellular matrix, and epithelial-mesenchymal transition of renal tubular cells [35]. In result of active inflammation, the tubulointerstitial tissue is infiltrated by leucocytes, including monocytes that differentiate into tissue macrophages and initiate the repair process and induce fibrosis. This may lead to an increase in the values of uNCR and/or uACR in some T2DM patients [36–38]. In our study, 20% of T2DM patients had elevated uNCR (above the maximum value in the control group), and less than half of those patients had elevated albuminuria. Fu et al. [14] observed that tubular damage defined by the increase in uNCR appears even in patients with diabetes of short duration, and the uNGAL may become a more promising and earlier marker of kidney damage in T2DM than uACR. Similarly, in the study by Kim et al. [15] nonalbuminuric proteinuria correlated significantly with uNGAL in patients with early-stage DKD (eGFR ≥ 60 ml/min/1.73 m^2^).

The majority of our patients were treated with angiotensin-converting enzyme inhibitors or angiotensin receptor blockers as part of nephroprotection in DKD [34]. We have not observed statistically significant differences in uNCR or uACR between patients treated with renin-angiotensin-aldosterone (RAA) system blockade and those not treated, although there was a tendency towards higher uNCR in patients who were on such treatment. These results may suggest poor protective effect of RAA blockade against tubular changes in T2DM. This observation is consistent with the results of Nielsen et al. [30] who reported no effects of angiotensin-converting enzyme inhibitors treatment on uNGAL concentration in patients with T2DM with normal glomerular filtration rate and “microalbuminuria.”

In the present study, patients with better diabetes control and less atherogenic lipid profile had also lower uNCR values. There is evidence that, in T2DM patients with CKD stages 1 to 4, better glycemic control contributes to improved kidney function and brings benefits to the vascular system [22, 39]. Also, some studies suggest that lowering total cholesterol slows down the progression of renal disease in T2DM [40, 41]. As uNCR value may be considered a noninvasive indicator of renal tubules' function, the results of the present study lead us to hypothesis that better diabetes control together with the treatment of dyslipidemia may have a positive influence on the tubule function and probably also renal interstitial changes. However, to validate this hypothesis, further prospective studies on a larger population of T2DM patients are required.

## 5. Conclusion

Combinations of biomarkers representing different mechanisms of DKD pathogenesis may be helpful in the determination of a pattern of changes in kidney function, especially in the heterogenic group of T2DM patients. Our results suggest that the determination of uNCR in addition to uACR and eGFR enables early detection of kidney disease in a part of patients with T2DM. Our results should be treated with caution, because of the limited numbers of T2DM patients and low number of controls recruited. However, our results suggest that uNCR values higher than a cut-off value (39.64 *µ*g/g in our sample) may be an indicator of early damage to renal tubules, especially in T2DM women with dyslipidemia and worse diabetes control. The results, especially the cut-off value, should be validated in larger study.

## Figures and Tables

**Table 1:** Characteristics of studied patients.

	Control patients (*N* = 22)	T2DM patients (*N* = 123)	*p* value
Age, years	57 ± 15	62 ± 13	0.1
Male gender, *N* (%)	9 (41)	57 (46)	0.7
BMI, kg/m^2^	28 ± 6	32 ± 6	0.009
eGFR, ml/min/1.73 m^2^	87 ± 15	90 ± 17	0.3
Hypertension, *N* (%)	15 (68)	98 (80)	0.2
Ischemic heart disease, *N* (%)	3 (14)	24 (19)	0.5
Heart failure, *N* (%)	3 (14)	9 (7)	0.3
Dyslipidemia, *N* (%)	19 (95)	111 (95)	1.0
Treatment with ACEI or ARB, *N* (%)	12 (55)	86 (70)	0.2
Urine albumin, mg/l	5.9 (3.0–22.9)	8.0 (3.2–18.0)	0.6
uACR, mg/g	6.0 (3.6–9.0)	7.3 (3.4–19.2)	0.3
Urine NGAL, *µ*g/l	10.9 (6.0–38.2)	15.3 (6.4–29.6)	0.7
uNCR, *µ*g/g	12.2 (5.9–27.9)	13.5 (6.5–31.4)	0.3

T2DM, type 2 diabetes mellitus; BMI, body mass index; eGFR, estimated glomerular filtration rate; ACEI, angiotensin-converting enzyme inhibitors; ARB, angiotensin receptor blockers; uACR, urine albumin/creatinine ratio; NGAL, neutrophil gelatinase-associated lipocalin; uNCR, urine NGAL/creatinine ratio.

**Table 2:** The differences between T2DM patients with urine NGAL to creatinine ratio (uNCR) below and above the maximum control value.

	T2DM patients with uNCR ≤ 39.64 *µ*g/g (*N* = 99)	T2DM patients with uNCR > 39.64 *µ*g/g (*N* = 24)	*p* value
Age, years	62 ± 12	62 ± 17	0.9

Men, *N* (%)	54 (55)	3 (12)	<0.001

BMI, kg/m^2^	32 ± 5	32 ± 7	0.9

Treatment with ACEI or ARB, *N* (%)	67 (68)	19 (79)	0.3

T2DM duration, years	6 (1–10)	6 (5–12)	0.5

Newly diagnosed diabetes, *N* (%)	27 (27)	4 (16)	0.3

Ophthalmologic examination, *N* (%)	83 (84)	11 (46)	<0.001
Retinopathy, *N* (% of examined)	17 (20)	2 (18)	0.9

HbA1c, %/mmol/mol	6.50 (5.90–8.50)/47.5 (41.0–69.4)	6.95 (6.10–8.60)/52.5 (43.2–70.5)	0.4

eGFR, ml/min/1.73 m^2^	90 ± 17	91 ± 20	0.9

Triglycerides, mmol/l	1.56 (1.20–2.03)	2.42 (1.40–3.39)	0.021

Total cholesterol, mmol/l	4.59 (3.83–5.72)	5.74 (4.55–7.14)	0.003

LDL-cholesterol, mmol/l	2.60 (1.95–3.61)	3.69 (2.40–4.47)	0.004

HDL-cholesterol, mmol/l	1.19 (0.96–1.46)	1.23 (1.06–1.40)	0.5

Urine albumin, mg/l	6.8 (3.0–12.9)	16.3 (10.7–35.9)	0.003

uACR, mg/g	4.8 (3.1–13.0)	16.0 (9.1–50.0)	<0.001

uACR < 30 mg/g, *N* (%)	84 (88)	13 (54)	<0.001

T2DM, type 2 diabetes mellitus; BMI, body mass index; eGFR, estimated glomerular filtration rate; ACEI, angiotensin-converting enzyme inhibitors; ARB, angiotensin receptor blockers; uACR, urine albumin/creatinine ratio; NGAL, neutrophil gelatinase-associated lipocalin; uNCR, urine NGAL/creatinine ratio.

**Table 3:** Multiple linear regression to predict uNCR > 39.64 *µ*g/g in patients with T2DM.

Dependent variables	Odds ratio (95% confidence interval)	*p* value
Women	7.98 (1.90–33.3)	0.004
Triglycerides, per 1 mmol/l	1.26 (0.73–2.16)	0.4
Total cholesterol, per 1 mmol/l	1.83 (1.15–2.91)	0.009
uACR, per 1 mg/g	1.03 (1.004–1.05)	0.022

T2DM, type 2 diabetes mellitus; uACR, urine albumin/creatinine ratio; uNCR, urine NGAL/creatinine ratio.
